# *Elizabethkingia* Infections in Humans: From Genomics to Clinics

**DOI:** 10.3390/microorganisms7090295

**Published:** 2019-08-28

**Authors:** Jiun-Nong Lin, Chung-Hsu Lai, Chih-Hui Yang, Yi-Han Huang

**Affiliations:** 1 School of Medicine, College of Medicine, I-Shou University, Kaohsiung 824, Taiwan; 2 Division of Infectious Diseases, Department of Internal Medicine, E-Da Hospital, I-Shou University, Kaohsiung 824, Taiwan; 3 Department of Critical Care Medicine, E-Da Hospital, I-Shou University, Kaohsiung 824, Taiwan; 4 Department of Biological Science and Technology, Meiho University, Pingtung 912, Taiwan

**Keywords:** *Elizabethkingia meningoseptica*, *Elizabethkingia miricola*, *Elizabethkingia anophelis*, *Elizabethkingia bruuniana*, *Elizabethkingia ursingii*, *Elizabethkingia occulta*, epidemiology, drug resistance, genomics

## Abstract

The genus *Elizabethkingia* has recently emerged as a cause of life-threatening infections in humans, particularly in immunocompromised patients. Several new species in the genus *Elizabethkingia* have been proposed in the last decade. Numerous studies have indicated that *Elizabethkingia anophelis*, rather than *Elizabethkingia meningoseptica*, is the most prevalent pathogen in this genus. Matrix-assisted laser desorption/ionization–time of flight mass spectrometry systems with an extended spectrum database could reliably identify *E. anophelis* and *E. meningoseptica*, but they are unable to distinguish the remaining species. Precise species identification relies on molecular techniques, such as housekeeping gene sequencing and whole-genome sequencing. These microorganisms are usually susceptible to minocycline but resistant to most β-lactams, β-lactam/β-lactam inhibitors, carbapenems, and aminoglycosides. They often exhibit variable susceptibility to piperacillin, piperacillin-tazobactam, fluoroquinolones, and trimethoprim-sulfamethoxazole. Accordingly, treatment should be guided by antimicrobial susceptibility testing. Target gene mutations are markedly associated with fluoroquinolone resistance. Knowledge on the genomic characteristics provides valuable insights into in these emerging pathogens.

## 1. Introduction

**Figure 1:**
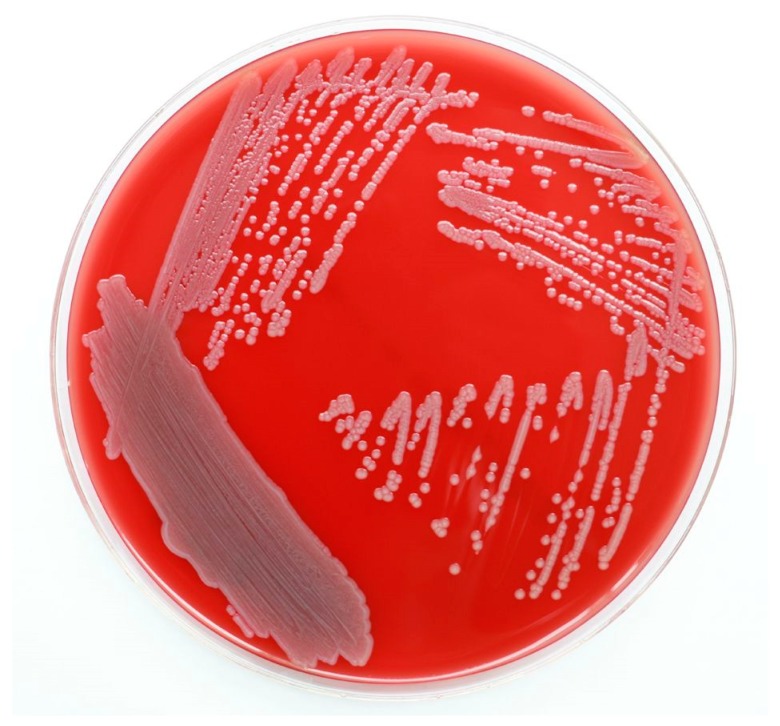
*Elizabethkingiameningoseptica* on sheep blood agar after 48 h of incubation.

Microorganisms in the genus *Elizabethkingia* are Gram-negative, aerobic, pale yellow-pigmented, nonmotile, glucose-non-fermenting, non-spore-forming, oxidase-positive, weakly indole-positive, and nitrate-negative bacilli (Figure 1) [1,2]. These bacteria are ubiquitously distributed in natural environments such as water, soils, fish, frogs, and insects [3,4,5,6,7,8], as well as in the tap water of hospitals [9,10,11]. Since its first identification in 1959 [12], *Elizabethkingia* has been occasionally reported to cause human infections. Recently, these bacteria have emerged as a major cause of life-threatening infections in numerous countries [13,14,15,16,17,18,19,20,21,22].

Along with advances in genetics and molecular technology, high-throughput next-generation sequencing has become a powerful tool in research and clinical medicine. Whole-genome sequencing has been widely used to investigate the genomic features, evolutionary relationship, epidemiology, species delineation, virulence factors, and antibiotic resistance of microorganisms, particularly in emerging pathogens [23,24]. Herein, we review the literature related to the genomic studies and the taxonomy, species identification, epidemiology, clinical characteristics, and antimicrobial susceptibility testing of *Elizabethkingia* infections in humans. This review provides insights into the genomics and clinics of this emerging infection.

## 2. Taxonomy and Nomenclature

In the 1950s, an increase in meningitis in infants caused by an unknown Gram-negative rod-shaped bacterium attracted attention in the United States. This unclassified bacterium was designated as group IIa by the US Centers for Disease Control and Prevention (CDC). In 1959, an American microbiologist at the CDC, Elizabeth O. King, investigated this pathogen and named it *Flavobacterium meningosepticum* (Table 1) [12]. This bacterium was moved to a new genus and renamed *Chryseobacterium meningosepticum* in 1994 [25]. Kim et al. proposed *Elizabethkingia* gen. nov. later, and *C. meningosepticum* was then renamed *Elizabethkingia meningoseptica* in 2005 [1].

In 2003, Li et al. identified a novel species, *Chryseobacterium miricola,* from condensation water collected in 1997 on the Mir space station of Russia [26]. This new species was assigned to the genus *Elizabethkingia* along with *E. meningoseptica* and was renamed *Elizabethkingia miricola* [1]. The third species, *Elizabethkingia anophelis*, was recovered from the midgut of *Anopheles gambiae* mosquitos in the Gambia, Africa, by Kämpfer et al. in 2011 [3]. Four years later, *Elizabethkingia endophytica* sp. nov. (type strain JM-87^T^) was proposed [27]. However, this strain was recognized as a later subjective synonym of *E. anophelis* as per the comparative genomics of whole-genome sequencing [28]. In August 2017, Nicholson et al. investigated bacteria of the unknown CDC genomospecies, and *Elizabethkingia bruuniana*, *Elizabethkingia ursingii*, and *Elizabethkingia occulta* were proposed as new members of the genus *Elizabethkingia* [2]. Currently, the genus *Elizabethkingia* comprises six species, namely, *E. meningoseptica*, *E. miricola*, *E. anophelis*, *E. bruuniana*, *E. ursingii*, and *E. occulta*.

## 3. Identification of Species

Accurately identifying offensive pathogens, either for clinical practice or basic research, is imperative. However, the identification of *Elizabethkingia* species remains a considerable challenge in clinical settings.

### 3.1. Biochemical-Based Phenotyping and Matrix-Assisted Laser Desorption/Ionization–Time of Flight Mass Spectrometry

Both biochemical-based phenotyping and matrix-assisted laser desorption/ionization–time of flight mass spectrometry (MALDI–TOF MS) systems are extensively used for microbial identification in clinical microbiology laboratories. The most widely used microbial identification systems include API/ID32 Phenotyping Kits (bioMérieux, Marcy l’Etoile, France), Phoenix 100 ID/AST Automated Microbiology System (Becton Dickinson Co., Sparks, MD, USA), Vitek 2 Automated Identification System (bioMérieux), Vitek MS (bioMérieux), and Bruker Biotyper MS (Bruker Daltonics GmbH, Bremen, Germany). However, these systems contain only a portion of *Elizabethkingia* species in their reference databases (Table 2). The newly proposed *Elizabethkingia* species are actually not included in the reference databases of these commercial identification systems. The lack of species information in the reference databases prevents these platforms from correctly recognizing the species of *Elizabethkingia*.

Several studies have demonstrated low reliability levels of common microbial identification systems in the identification of *Elizabethkingia* species. Lin et al. compared the accuracy of API/ID32, Phoenix 100 ID/AST, Vitek 2, and Vitek MS with that of 16S ribosomal RNA (rRNA) gene sequencing for the identification of 49 *Elizabethkingia* isolates in Taiwan [29]. The concordances of species identification between these machines and 16S rRNA gene sequencing were only 24.5%–26.5%. Lau et al. retrospectively analyzed 21 *Elizabethkingia* isolates in Hong Kong [14], namely 17 *E. anophelis*, three *E. meningoseptica*, and one *E. miricola*, as determined through 16S rRNA gene sequencing. All isolates were identified as *E. meningoseptica* by Vitek 2; the 17 *E. anophelis* were misidentified as *E. meningoseptica* or unidentified by the Bruker Biotyper equipped with a default spectrum library. In another retrospective study performed in South Korea [30], Han et al. investigated 51 *E. anophelis*, 17 *E. meningoseptica*, and 18 *E. miricola* species. Similar to the report of Lau et al. [14], *E. meningoseptica* could be accurately identified by Vitek 2, Vitek MS, and Bruker Biotyper, but almost all *E. anophelis* species were misidentified as *E. meningoseptica* by Vitek 2 and MALDI–TOF MS with a default database.

Although MALDI–TOF MS systems equipped with commercial reference databases cannot recognize *E. anophelis*, systems with amended databases—such as the “research-use-only” (Saramis) database of Vitek MS [30,31], in-house expanded spectrum database of Bruker Biotyper [14], and expanded spectral library provided by the CDC Special Bacteriology Reference Laboratory of Bruker Biotyper [15]—could reliably distinguish *E. anophelis* from *E. meningoseptica*. Cheng et al. investigated the specific peaks of each *Elizabethkingia* species using the Vitek MS research-use-only system [31]. Some specific mass-to-charge ratio (*m/z*) values, namely, peaks at 7643.7/10320.9 *m/z* in *E. anophelis*, 3141.5/12109.1 *m/z* in *E. meningoseptica*, and 3792.5/7586.6 *m/z* in *E. miricola* cluster, were observed. These specific peaks in MALDI–TOF MS could be used to differentiate *Elizabethkingia* species. However, these amended databases, either in the Vitek MS or Bruker Biotyper systems, are primarily available for research purposes but are not for clinical application in clinical microbiology laboratories. Additionally, although MALDI–TOF MS systems with expanded spectrum databases could reliably identify *E. anophelis* and *E. meningoseptica*, these platforms cannot distinguish between the remaining species of the genus *Elizabethkingia* [2,31].

### 3.2. Housekeeping Gene Sequencing

Housekeeping gene sequencing has been increasingly used for microbial identification. Among the genotyping techniques of housekeeping gene sequencing, 16S rRNA and RNA polymerase β-subunit (*rpoB*) gene sequencing are the two most commonly used methods for microbial identification [32,33].

In the literature, 16S rRNA gene sequencing is considered an accurate method for identifying *Elizabethkingia* species [34]. The total length of the 16S rRNA gene in *Elizabethkingia* species is 1521 bp [14,18,19]. The 16S rRNA gene includes nine hypervariable regions V1–V9 and these regions possess varying lengths and conservation in different bacterial species. Some of the hypervariable regions exhibit more variabilities than others [32]. However, no studies to date investigate the 16S rRNA hypervariable regions in *Elizabethkingia* species. Moreover, the existence of multiple 16S rRNA copies with different sequences in bacteria has advanced the argument for the use of 16S rRNA gene sequencing in microbial identification [35]. For example, five 16S rRNA gene copies in an *E. ursingii* strain were discovered; one 16S rRNA gene was similar to the *E. ursingii* type strain G4122^T^, two were similar to *E. bruuniana*, and the remaining two matched each other but were otherwise unique [2]. Therefore, species identification using 16S rRNA gene sequencing could be misleading.

*rpoB* is highly conserved in microorganisms and has become a potential candidate for microbial identification [36,37]. *rpoB* typically presents with a single copy, and it possesses a higher resolution of phylogenetic evolution than does the 16S rRNA gene [33]. A molecular phylogenetic analysis of *rpoB* at positions 1939–3629 was proved to be able to correctly distinguish *Elizabethkingia* strains at the species level [2]. However, studies have yet to compare the accuracy of 16S rRNA and *rpoB* gene sequencing in identifying species in the genus *Elizabethkingia*.

### 3.3. Polymerase Chain Reaction Assay

Two polymerase chain reaction (PCR)-based methods have been recently developed for detecting and differentiating *Elizabethkingia* [38,39]. After a comparative analysis of the whole-genome sequences of different *Elizabethkingia* species, species-specific genes in *E. anophelis* (encoding lipid A-disaccharide synthase) and *E. meningoseptica* (a putative gene of sodium-proton antiporter) were explored. The amplicon sizes for the differentiation of *E. anophelis* and *E. meningoseptica* were 281 bp and 250 bp, respectively. This method could clearly discriminate *E. anophelis* from *E. meningoseptica* with no cross-reactivity [38]. In addition, a multiplex real-time PCR technique was developed for detecting *Elizabethkingia* bacteria directly from primary specimens and discriminating *E. anophelis* and *E. meningoseptica* [39]. The highly conserved *secY* gene (PCR amplicon size, 146 bp) in all species was selected as a representative target of the genus *Elizabethkingia*. The elongation factor 4 *lepA* gene (PCR amplicon size, 142 bp) and phenylalanine-tRNA ligase β-subunit *pheT* gene (PCR amplicon size, 90 bp) were chosen to discriminate *E. anophelis* from *E. meningoseptica* [39]. These PCR assays emphasise the rapid and reliable recognition of the presence of *Elizabethkingia* species, and they simultaneously distinguish *E. anophelis* from *E. meningoseptica* [39].

### 3.4. Species Delineation through Whole-Genome Sequencing

DNA–DNA hybridization (DDH) has clearly served as the gold standard for distinguishing bacterial species since the 1960s [40]. However, conventional DDH is a complex and time-consuming procedure and is usually available only in some laboratories. With the introduction of high-throughput next-generation sequencing, whole-genome sequencing has been increasingly used in the species delineation of microorganisms [23,24]. Whole-genome sequence-based average nucleotide identity (ANI) analysis and in silico DDH are widely acknowledged as the two robust measures of genomic similarity between different strains [23,24,41,42]. These two methods have been demonstrated to yield a higher correlation than conventional DDH in the delineation of prokaryotic species [23,24,41,42]. Despite these merits, whole-genome sequencing remains a highly expensive and time-consuming procedure. Thus, the application of whole-genome sequencing in species delineation is usually limited in research.

Whole-genome sequencing is valuable in taxonomy for determining the relatedness of different species [2,28]. A number of whole-genome sequences of *Elizabethkingia* species are available in the GenBank of the National Center for Biotechnology Information [2,28,43,44,45,46,47]. The complete genome size of *Elizabethkingia* species is approximately 4.3–4.4 Mbp, and contains approximately 4000 coding sequences [2,3,27,43,44,45,46,47]. As mentioned, *E. endophytica* was proposed as a novel species in the genus *Elizabethkingia* [27]. However, the in silico DDH and ANI values between the *E. endophytica* type strain JM87^T^ and *E. anophelis* type strain R26^T^ were 77% (cutoff value of species delimitation, 70%) and 97% (cutoff value of species delimitation, 95%), respectively [28]. This finding suggests that *E. endophytica* actually represents a strain of *E. anophelis* and not a new species. In addition, substantial sequence variability in the whole-genome sequences of *E. miricola* strains has been observed [28,45]. According to the results of in silico DDH and ANI analyses, some strains of *E. miricola*, including ATCC 33958, BM10, and EM798-26, have been reassigned as *E. bruuniana* [2,43,46].

## 4. Epidemiology, Clinical Characteristics, and Outcomes

### 4.1. E. meningoseptica

As mentioned, several *Elizabethkingia* species were misidentified as *E. meningoseptica*. Therefore, previous studies on the epidemiology and clinical features of *E. meningoseptica* could have substantial bias if they were performed before the proposal of novel species or species identification relying on inaccurate methods [48,49,50,51,52,53,54,55,56,57].

The incidence of *E. meningoseptica* infection is not entirely clear. A South Korean study reported that the annual incidence of *E. meningoseptica* infection was 0.01 per 1000 admissions in 2009 and then increased to 0.04 per 1000 admissions from 2016–2017 [17]. Actually, *E. meningoseptica* only accounted for approximately 1%–21% of all *Elizabethkingia* pathogens isolated from clinical specimens. By contrast, *E. anophelis* was the most prevalent pathogen in this genus, constituting 59%–99% of all isolates [14,17,18,30].

The majority of *E. meningoseptica* infections present as meningitis, bacteremia, pneumonia, skin and soft-tissue infection, catheter-associated infection, and urinary tract infection in neonates, infants, and immunocompromised patients [49,50,51,52,58,59]. Most cases were attributed to health care-associated infections, and over 85% of patients had at least one comorbidity [19]. The case-fatality rate of *E. meningoseptica*-infected patients has been reported to be 30%–54% [9,19]. An epidemiologic investigation revealed that water is a transmission route of *E. meningoseptica* infection in hospitals [9].

### 4.2. E. miricola

*E. miricola* has been sporadically reported to cause pneumonia, bacteremia, urinary tract infection, and periodontitis since its first proposal as a new species in 2003 [60,61,62,63]. A retrospective South Korean study reported that the annual incidence of *E. miricola* increased from 0 to 0.22 per 1000 admissions from 2009–2016 [17]. Nevertheless, the prevalence of *E. miricola* markedly varies in different geographic areas [14,29,31,38]. A Singapore study retrospectively analyzed 79 bacteremic *Elizabethkingia* isolates collected from 2009–2019, and no *E. miricola* was identified using 16S rRNA gene sequencing [38]. Studies in Hong Kong [14] and Taiwan [29] have reported that only one *E. anophelis* isolate was recognized from a collection of 21 and 49 *Elizabethkingia* isolates, respectively. However, another study performed in northern Taiwan discovered 18 isolates of *E. miricola* among 269 *Elizabethkingia* isolates through *rpoB* sequencing [31]. Recently, *E. miricola* appears to have become a vital opportunistic pathogen in patients with cystic fibrosis in the United Kingdom [64]. Kenna et al. reported that 43 isolates from 38 patients with cystic fibrosis congregated in a cluster that shared >99% *rpoB* sequence similarity with the type strains of *E. miricola* and *E. bruuniana*. However, the species in this “*E. miricola* and *E. bruuniana* cluster” could not be precisely distinguished in that study [64].

### 4.3. E. anophelis

The first case of *E. anophelis* infection was reported in a neonatal meningitis patient in the Central African Republic in 2011 [13]. Thereafter, several studies have demonstrated that *E. anophelis* has recently emerged as a life-threatening infection in Singapore [11], Hong Kong [14], the United States [15,16,20,21], South Korea [17], and Taiwan [18]. However, as mentioned, *E. anophelis* is usually misidentified as *E. meningoseptica* by microbial identification platforms in clinical settings. Therefore, the incidence of *E. anophelis* infection could be substantially underestimated.

Several outbreaks of *E. anophelis* infection in humans have been described. In 2012, an outbreak occurred in five patients in an intensive care unit in Singapore, resulting in two deaths due to sepsis [11]. The largest outbreak to date was described in the Midwestern United States—Wisconsin, Illinois, and Michigan—from 2015–2017 [15,16,20,21]. A total of 63 patients were confirmed to have *E. anophelis* infection in Wisconsin, and this outbreak caused 19 deaths [20]. Another outbreak affecting ten patients occurred in Illinois, and six of these patients died of this infection [21]. Despite these outbreak announcements, the actual prevalence of *E. anophelis* infection is unclear. Only one study in South Korea reported that the annual incidence of *E. anophelis* infection increased from 0.01 to 0.6 per 1000 admissions from 2009–2017 [17].

The transmission route of *E. anophelis* remains unclear. *E. anophelis* is suspected to be transmitted by an insect vector considering that *E. anophelis* was originally discovered in the *A. gambiae* mosquito [3]. However, currently, evidence supporting that *E. anophelis* infection is a mosquito-borne disease is unavailable. Investigations of the infection source in the Midwest outbreak remain undetermined despite aggressive examinations of tap water, food, and personal hygiene products [15,16,20,21]. Perinatal vertical transmission from mother to infant has been established in Hong Kong through a whole-genome analysis of the offending strains [65]. In addition, an epidemiologic investigation of outbreaks in Singapore revealed *E. anophelis* in the tap water aerators of the hospital [10,11]. *E. anophelis* was suggested to be transmitted from the hands of health care workers, who acquired this bacterium during handwashing, to patients. The removal of the aerators and the use of alcohol-based hand rubs after hand hygiene could effectively eliminate the transmission of *E. anophelis* [10].

The clinical presentations of *E. anophelis* infections are protean, including bacteremia, pneumonia, catheter-related bloodstream infection, meningitis, skin and soft-tissue infection, urinary tract infection, and biliary tract infection [14,19,20,21]. Most infections (80%–87.5%) are hospital-acquired [14,19]. However, 89% of cases in the Wisconsin outbreak were attributed to community-onset infection [15,16,20,21]. Patients with *E. anophelis* infection are usually over 60 years old, and over 85% of patients have comorbidities, such as diabetes mellitus, malignancy, chronic renal disease, end-stage renal disease with dialysis therapy, liver cirrhosis, alcohol dependence, immune-compromising conditions, and receiving immunosuppressive treatment [14,15,16,17,18,19,20,21]. The case-fatality rate of patients with *E. anophelis* infection is critically high, ranging from 24% to 60% [14,15,16,17,18,19,20,21]. Notably, inappropriate empirical antimicrobial therapy is an independent risk factor for mortality in patients infected with E. anophelis [18,19].

### 4.4. Other Elizabethkingia Species

After the proposal of *E. bruuniana*, *E. ursingii*, and *E. occulta*, only a few studies have discussed these novel species. In the aforementioned study on patients with cystic fibrosis in the United Kingdom [64], one isolate expressed 99.3% similarity of *rpoB* with the type strain of *E. ursingii*, and 43 isolates formed an ‘*E. miricola* and *E. bruuniana* cluster’. However, *E. bruuniana* could not be differentiated from *E. miricola*. A study from Taiwan retrospectively analyzed 269 *Elizabethkingia* isolates by using 16S rRNA and *rpoB* sequencing, and one *E. bruuniana* and two *E. occulta* were recognized [31]. Another study published the complete whole-genome sequence of the *E. miricola* strain EM798-26, which was isolated from the blood of an 81-year-old male patient with diffuse large B-cell lymphoma in Taiwan [46]. After a comprehensive genomic investigation, this strain was amended as *E. bruuniana*. Recently, six patients with health care-associated *E. bruuniana* infections were reported in Taiwan using 16S rRNA and *rpoB* gene sequencing [66]. The isolation sources of *E. bruuniana* included blood, bronchoalveolar lavage fluid, urine, and the tip of the central venous catheter. None of the patients died of E. bruuniana infection [66].

## 5. Antimicrobial Susceptibility Testing and Antibiotic Resistance Genes

The antimicrobial susceptibility patterns of *Elizabethkingia* reported in the literature are summarized in Table 3. Notably, the antibiotic susceptibilities, particularly for vancomycin and piperacillin-tazobactam, determined by the disk diffusion test, E-test assay, and agar dilution test are considered to be unreliable and inaccurate for *Elizabethkingia* species [67]. The broth microdilution test is recommended for susceptibility determination. However, only two studies from Taiwan [19,31] and one from Singapore [38] have employed the broth microdilution method to determine the antibiotic susceptibility for *Elizabethkingia* species.

### 5.1. E. meningoseptica

Most studies investigating the antimicrobial resistance of *E. meningoseptica* were performed before the proposal of *E. anophelis*. As mentioned, these studies have actually represented the antimicrobial susceptibility patterns of all *Elizabethkingia* species, particularly *E. anophelis*, but not those of *E. meningoseptica*. Currently, only a few studies have examined the antimicrobial susceptibility of a collection of *E. meningoseptica* by using reliable species identification methods (Table 3).

Studies have revealed that *E. meningoseptica* isolates were usually resistant to cephalosporins, carbapenems, and aminoglycosides [19,30,31]. These isolates displayed variable susceptibility levels to piperacillin (15%–65%), piperacillin-tazobactam (5%–100%), ciprofloxacin (10%–23%), and levofloxacin (30%–55%). The rate of susceptibility to trimethoprim-sulfamethoxazole was relatively low (6%–10%). Most isolates (60%–100%) were susceptible to minocycline. Notably, no *E. meningoseptica* was susceptible to vancomycin. Although some anecdotal reports have revealed the successful treatment of *E. meningoseptica* meningitis using a combination therapy of vancomycin with other antibiotics [68], the use of vancomycin is not suggested because of its high minimum inhibitory concentration [19,30].

Whole-genome research conducted on *E. meningoseptica* has identified numerous putative genes conferring antibiotic resistance [19,69]. However, only a few homologs have been further investigated. Two recent studies have explored the association between fluoroquinolone resistance and target gene mutations in *E. meningoseptica* [19,70]. Several point mutations were detected in the quinolone-resistance-determining regions (QRDRs) of DNA gyrase subunit A (GyrA) and subunit B (GyrB). Amino acid alterations Ser83Ile/Pro95Ser in GyrA and Ser452Arg/Glu470Asp in GyrB were significantly associated with levofloxacin resistance [19,70].

### 5.2. E. miricola

Similar to *E. meningoseptica*, *E. miricola* is usually resistant to multiple antibiotics (Table 3). In analyses of 18 isolates collected in South Korea [30] and 22 isolates obtained in Taiwan [31], all *E. miricola* isolates were resistant to cephalosporins, aminoglycosides, and carbapenems. These isolates were most susceptible to minocycline (100%), levofloxacin (77%–100%), piperacillin-tazobactam (73%–94%), piperacillin (83%), rifampin (66%), and ciprofloxacin (14%–56%).

A Switzerland study recognized genes encoding metallo-β-lactamases (BlaB-15 and a GOB-7-like enzyme) in a multidrug-resistant *E. miricola* isolated from the urine of a 2-year-old boy [61]. These genes confer resistance to penicillin–β-lactamase inhibitor combinations, cefotaxime, cefoxitin, and carbapenems. Another Switzerland study reported a carbapenemase-producing clinical isolate of *E. miricola* EM_CHUV recovered from the lower respiratory tract specimen of a patient with severe nosocomial pneumonia [62]. This strain was sensitive to gentamicin, amikacin, and levofloxacin but resistant to all tested β-lactams, β-lactam/β-lactams inhibitor combinations, ciprofloxacin, and carbapenems. Whole-genome analysis disclosed the presence of numerous antibiotic resistance genes, including *bla*_GOB-13_ and *bla*_B-9_ encoding for class B carbapenemases. In addition, the amino acid alterations Ser83Ile/Asp87Asn/Thr83Ser in GyrA, Met437Leu in GyrB, and Met437Phe/Ala473Leu in ParE have been detected in some studies [62,70]. The presence of abundant antibiotic resistance genes is compatible with multidrug resistance.

### 5.3. E. anophelis

Several studies have demonstrated that *E. anophelis* isolates were resistant to most β-lactams, β-lactam/β-lactam inhibitor combinations, carbapenems, and aminoglycosides [14,15,19,30,31,38] (Table 3). Nevertheless, over 90% of *E. anophelis* isolates in the Wisconsin outbreak were remarkably susceptible to cefepime [15]. A wide variation exists in the susceptibility of *E. anophelis* to piperacillin (19.5%–100%), piperacillin-tazobactam (30.6%–92%), ciprofloxacin (1%–100%), levofloxacin (16%–96%), trimethoprim-sulfamethoxazole (4%–70.6%), and vancomycin (0%–100%), whereas almost all *E. anophelis* isolates were notably susceptible to minocycline (97.5%–100%). It is noteworthy that different testing methods could cause the variations in the susceptibility patterns observed among these studies, apart from the geographic variation.

Currently, over 60 deposited whole-genome sequences of *E. anophelis* are available in the GenBank. Whole-genome studies have revealed numerous putative genes associated with antibiotic resistance in *E. anophelis* [15,44,47,71]. These antimicrobial resistance-associated genes comprise genes conferring resistance to β-lactams (such as *bla*_CME-1_, *bla*_bla*B*_, and *bla*_GOB-4_), aminoglycosides, fluoroquinolones, tetracycline, macrolides, chloramphenicol, vancomycin, trimethoprim, and multidrug resistance efflux pumps. Among these antibiotic resistance genes, mutations in the fluoroquinolone target genes have been well investigated. Investigations of *E. anophelis* isolates from the Wisconsin outbreak and from Taiwan have revealed that amino acid alterations at position 83 (Ser83Ile/Ser83Arg) and position 95 (Pro95Ser) in GyrA were associated with high-level fluoroquinolone resistance [15,18,19,70,72]. No nonsynonymous substitutions were recognized in GyrB, ParC, and ParE. Furthermore, gene mutations in the QRDRs occurred considerably less frequently in *E. anophelis* than they did in *E. meningoseptica* [19]. This finding is consistent with the fact that *E. anophelis* usually exhibited a substantially higher susceptibility rate to levofloxacin than *E. meningoseptica* did [19].

### 5.4. Other Elizabethkingia Species

Limited information presently exists regarding the antimicrobial susceptibility of the recently proposed *E. bruuniana*, *E. ursingii*, and *E. occulta*. A recent study described six *E. bruuniana* strains isolated from a clinical specimen in Taiwan [66]. Most isolates were resistant to β-lactams, β-lactam and lactamase inhibitors, carbapenems, aminoglycosides, and trimethoprim/sulfamethoxazole, but all these isolates were susceptible to minocycline. Two-thirds of isolates were susceptible to levofloxacin. However, no nonsynonymous substitutions in the QRDRs of *gyrA*, *gyrB*, *parC*, and *parE* were identified [66]

## 6. Conclusions

*Elizabethkingia*, particularly *E. anophelis*, has rapidly spread in several countries and causes lethally opportunistic infections in patients. Because numerous studies and clinical practice continue to rely on automated bacteriology identification systems for the identification of *Elizabethkingia*, upgrading MALDI–TOF MS with expanded reference databases or using molecular techniques to accurately identify these microorganisms is imperative. Considering that *Elizabethkingia* species are usually resistant to multiple antibiotics and that inappropriate antimicrobial therapy is an independent risk factor for mortality, early diagnosis and adequate antibiotic treatment are vital for patients with *Elizabethkingia* infection. *Elizabethkingia* demonstrates variable susceptibility to multiple antibiotics; therefore, treatment would be more reliable if guided by antimicrobial susceptibility testing. Minocycline has the potential to be the drug of choice for patients with *Elizabethkingia* infection. However, there is still a lack of clinical trials. Additional studies are required to determine optimal antimicrobial agents, either singly or in combination, for these life-threatening infections.

## Figures and Tables

**Table 1:** Taxonomy and nomenclature of *Elizabethkingia* species.

Present Name	History of Nomenclature	Author (Year)	Source of Isolation	Reference
*Elizabethkingia meningoseptica*	*Flavobacterium meningosepticum*	King (1959)	Cerebrospinal fluid, blood, throat of infants	[12]
	*Chryseobacterium meningosepticum*	Vandamme et al. (1994)		[25]
	*Elizabethkingia meningoseptica*	Kim et al. (2005)		[1]
*Elizabethkingia miricola*	*Chryseobacterium miricola*	Li et al. (2003)	Condensation water on Mir space station collected in 1997	[26]
	*Elizabethkingia miricola*	Kim et al. (2005)		[1]
*Elizabethkingia anophelis*	*Elizabethkingia anophelis*	Kämpfer et al. (2011)	The midgut of *Anopheles gambiae* in the Gambia, Africa	[3]
*Elizabethkingia bruuniana*	*Elizabethkingia bruuniana*	Nicholson et al. (2017)	Centers for Disease Control and Prevention (CDC) genomospecies 3	[2]
*Elizabethkingia ursingii*	*Elizabethkingia ursingii*	Nicholson et al. (2017)	CDC genomospecies 4	[2]
*Elizabethkingia occulta*	*Elizabethkingia occulta*	Nicholson et al. (2017)	Novel CDC genomospecies	[2]

**Table 2:** The coverage of the reference database of five common commercial microbial identification systems.

Species	API/ID32 v3.1	Phoenix 100 ID/AST v5.51A	Vitek 2 v7.01	Vitek MS Knowledge Base v2.0/v3.0	Bruker Biotyper Reference Library v6.0 6903
*E. meningoseptica*	Yes	Yes	Yes	Yes	Yes
*E. miricola*	No	Yes	No	No	Yes
*E. anophelis*	No	No	No	No	No
*E. bruuniana*	No	No	No	No	No
*E. ursingii*	No	No	No	No	No
*E. occulta*	No	No	No	No	No

**Table 3:** The antibiotic susceptible rate of *Elizabethkingia* species.*.

Antimicrobial Agent	*E. meningoseptica*			*E. miricola*		*E. anophelis*					
	Han et al. [30] *n* = 17	Cheng et al. [31] *n* = 11	Lin et al. [19] *n* = 20	Han et al. [30] *n* = 18	Cheng et al. [31] *n* = 22 ^†^	Lau et al. [14] *n* = 17	Han et al. [30] *n* = 51	Perrin et al. [15] *n* = 25	Cheng et al. [31] *n* = 105	Chew et al. [38] *n* = 79 ^‡^	Lin et al. [19] *n* = 72
	South Korea	Taiwan	Taiwan	South Korea	Taiwan	Hong Kong	South Korea	USA	Taiwan	Singapore	Taiwan
Piperacillin	65	-	15	83	-	41·1	82	100	-	-	19·4
Piperacillin-tazobactam	100	73	5	94	73	-	92	92	73	92·4	30·6
Ticarcillin-clavulanic acid	-	0	0	-	0	-	-	-	0	21.5	0
Cefoperazone-sulbactam	-	-	-	-	-	100	-	-	-	-	-
Ceftazidime	0	0	0	0	0	5·9	0	0	0	0	0
Cefepime	-	0	0	-	9	-	-	92	4	0	2·8
Ceftriaxone	-	-	0	-	-	-	-	-	-	-	0
Aztreonam	-	0	0	-	0	-	-	-	0	1·3	0
Ertapenem	-	0	0	-	0	-	-	-	0	-	0
Imipenem	0	0	0	0	0	0	0	0	0	0	0
Meropenem	-	0	0	-	0	-	-	-	0	0	0
Doripenem	-	0	0	-	0	-	-	-	0	0	0
Gentamicin	6	0	0	45	0	0	22	0	0	1·3	0
Tobramycin	-	0	0	-	0	0	-	0	0	0	0
Amikacin	-	0	0	-	9	0	-	0	0	6·3	5·6
Tetracycline	-	-	0	-	-	-	-	-	-	-	0
Minocycline	-	100	60	-	100	-	-	-	98	97·5	100
Doxycycline	-	91	-	-	82	-	-	-	83	92·4	-
Tigecycline	-	55	15	-	50	-	-	-	20	5·1	26·4
Ciprofloxacin	23	0	10	56	14	100	22	92	1	21·5	9·7
Levofloxacin	35	55	30	100	77	-	29	96	16	78·5	58·3
Moxifloxacin	41	-	-	100	-	-	41	-	-	-	-
Gatifloxacin	35	-	-	100	-	-	33	-	-	-	-
Trimethoprim-sulfamethoxazole	6	0	10	28	18	70·6	22	-	4	92·4	12·5
Rifampin	94	-	-	66	-	58·8	96	-	-	-	-
Vancomycin	0	-	0	0	-	100	0	-	-	-	0

* Methods of minimum inhibitory concentration determination: Han et al. [30], agar dilution test; Cheng et al. [31], broth microdilution test; Lin et al. [19], broth microdilution test; Lau et al. [14], disk diffusion test; Perrin et al. [15], disk diffusion test; and Chew et al. [38], broth microdilution test. ^†^ Representing an “*E. miricola* cluster” that contains *E. miricola*, *E. bruuniana*, *E. ursingii*, and *E. occulta*. ^‡^ Containing one *E. meningoseptica* and 78 *E. anophelis* isolates.

## References

[1] Kim K.K., Kim M.K., Lim J.H., Park H.Y., Lee S.-T. (2005). Transfer of *Chryseobacterium meningosepticum* and *Chryseobacterium miricola* to *Elizabethkingia* gen. nov. as *Elizabethkingia meningoseptica* comb. nov. and *Elizabethkingia miricola* comb. nov. Int. J. Syst. Evol. Microbiol..

[2] Nicholson A.C., Gulvik C.A., Whitney A.M., Humrighouse B.W., Graziano J., Emery B., Bell M., Loparev V., Juieng P., Gartin J. (2018). Revisiting the taxonomy of the genus *Elizabethkingia* using whole-genome sequencing, optical mapping, and MALDI-TOF, along with proposal of three novel *Elizabethkingia* species: *Elizabethkingia bruuniana* sp. nov., *Elizabethkingia ursingii* sp. nov., and *Elizabethkingia occulta* sp. nov. Antonie Van Leeuwenhoek.

[3] Kämpfer P., Matthews H., Glaeser S.P., Martin K., Lodders N., Faye I. (2011). *Elizabethkingia anophelis* sp. nov., isolated from the midgut of the mosquito *Anopheles gambiae*. Int. J. Syst. Evol. Microbiol..

[4] Jacobs A., Chenia H.Y. (2011). Biofilm formation and adherence characteristics of an *Elizabethkingia meningoseptica* isolate from *Oreochromis mossambicus*. Ann. Clin. Microbiol. Antimicrob..

[5] Mee P.T., Lynch S.E., Walker P.J., Melville L., Duchemin J.-B. (2017). Detection of *Elizabethkingia* spp. in *Culicoides* biting midges, Australia. Emerg. Infect. Dis..

[6] Hu R., Yuan J., Meng Y., Wang Z., Gu Z. (2017). Pathogenic *Elizabethkingia miricola* infection in cultured black-spotted frogs, China, 2016. Emerg. Infect. Dis..

[7] Lei X.P., Yi G., Wang K.Y., OuYang P., Chen D.F., Huang X.L., Huang C., Lai W.M., Zhong Z.J., Huo C.L. (2019). *Elizabethkingia miricola* infection in Chinese spiny frog (*Quasipaa spinosa*). Transbound. Emerg. Dis..

[8] Jiang H.-Y., Ma J.-E., Li J., Zhang X.-J., Li L.-M., He N., Liu H.-Y., Luo S.-Y., Wu Z.-J., Han R.-C. (2017). Diets alter the gut microbiome of crocodile lizards. Front. Microbiol..

[9] Moore L.S.P., Owens D.S., Jepson A., Turton J.F., Ashworth S., Donaldson H., Holmes A.H. (2016). Waterborne *Elizabethkingia meningoseptica* in adult critical care. Emerg. Infect. Dis..

[10] Yung C.-F., Maiwald M., Loo L.H., Soong H.Y., Tan C.B., Lim P.K., Li L., Tan N.W., Chong C.-Y., Tee N. (2018). *Elizabethkingia anophelis* and association with tap water and handwashing, Singapore. Emerg. Infect. Dis..

[11] Teo J., Tan S.Y.-Y., Tay M., Ding Y., Kjelleberg S., Givskov M., Lin R.T.P., Yang L. (2013). First case of *E anophelis* outbreak in an intensive-care unit. Lancet..

[12] King E.O. (1959). Studies on a group of previously unclassified bacteria associated with meningitis in infants. Am. J. Clin. Pathol..

[13] Frank T., Gody J.C., Nguyen L.B.L., Berthet N., Le Fleche-Mateos A., Bata P., Rafaï C., Kazanji M., Breurec S. (2013). First case of *Elizabethkingia anophelis* meningitis in the Central African Republic. Lancet.

[14] Lau S.K.P., Chow W.-N., Foo C.-H., Curreem S.O.T., Lo G.C.-S., Teng J.L.L., Chen J.H.K., Ng R.H.Y., Wu A.K.L., Cheung I.Y.Y. (2016). *Elizabethkingia anophelis* bacteremia is associated with clinically significant infections and high mortality. Sci. Rep..

[15] Perrin A., Larsonneur E., Nicholson A.C., Edwards D.J., Gundlach K.M., Whitney A.M., Gulvik C.A., Bell M.E., Rendueles O., Cury J. (2017). Evolutionary dynamics and genomic features of the *Elizabethkingia anophelis* 2015 to 2016 Wisconsin outbreak strain. Nat. Commun..

[16] Navon L., Clegg W.J., Morgan J., Austin C., McQuiston J.R., Blaney D.D., Walters M.S., Moulton-Meissner H., Nicholson A. (2016). Notes from the field: Investigation of *Elizabethkingia anophelis* cluster-Illinois, 2014–2016. MMWR.

[17] Choi M.H., Kim M., Jeong S.J., Choi J.Y., Lee I.-Y., Yong T.-S., Yong D., Jeong S.H., Lee K. (2019). Risk Factors for *Elizabethkingia* acquisition and clinical characteristics of patients, South Korea. Emerg. Infect. Dis..

[18] Lin J.-N., Lai C.-H., Yang C.-H., Huang Y.-H., Lin H.-H. (2018). Clinical manifestations, molecular characteristics, antimicrobial susceptibility patterns and contributions of target gene mutation to fluoroquinolone resistance in *Elizabethkingia anophelis*. J. Antimicrob. Chemother..

[19] Lin J.-N., Lai C.-H., Yang C.-H., Huang Y.-H. (2018). Comparison of clinical manifestations, antimicrobial susceptibility patterns, and mutations of fluoroquinolone target genes between *Elizabethkingia meningoseptica* and *Elizabethkingia anophelis* isolated in Taiwan. J. Clin. Med..

[20] Elizabethkingia. https://www.dhs.wisconsin.gov/disease/elizabethkingia.htm.

[21] Recent Outbreaks, *Elizabethkingia*, CDC. https://www.cdc.gov/elizabethkingia/outbreaks/.

[22] Snesrud E., McGann P., Walsh E., Ong A., Maybank R., Kwak Y., Campbell J., Jones A., Vore K., Hinkle M. (2018). Clinical and genomic features of the first cases of *Elizabethkingia anophelis* infection in New York, including the first case in a healthy infant without previous nosocomial exposure. J. Pediatr. Infect. Dis. Soc..

[23] Meier-Kolthoff J.P., Auch A.F., Klenk H.-P., Göker M. (2013). Genome sequence-based species delimitation with confidence intervals and improved distance functions. BMC Bioinformatics.

[24] Varghese N.J., Mukherjee S., Ivanova N., Konstantinidis K.T., Mavrommatis K., Kyrpides N.C., Pati A. (2015). Microbial species delineation using whole genome sequences. Nucleic Acids Res..

[25] Vandamme P., Bernardet J.-F., Segers P., Kersters K., Holmes B. (1994). New perspectives in the classification of the *Flavobacteria*: Description of *Chryseobacterium* gen. nov., *Bergeyella* gen. nov., and *Empedobacter* nom. rev. Int. J. Syst. Evol. Microbiol..

[26] Li Y., Kawamura Y., Fujiwara N., Naka T., Liu H., Huang X., Kobayashi K., Ezaki T. (2003). *Chryseobacterium miricola* sp. nov., a novel species isolated from condensation water of space station Mir. Syst. Appl. Microbiol..

[27] Kämpfer P., Busse H.-J., McInroy J.A., Glaeser S.P. (2015). *Elizabethkingia endophytica* sp. nov., isolated from Zea mays and emended description of *Elizabethkingia anophelis* Kämpfer et al. 2011. Int. J. Syst. Evol. Microbiol..

[28] Doijad S., Ghosh H., Glaeser S., Kämpfer P., Chakraborty T. (2016). Taxonomic reassessment of the genus *Elizabethkingia* using whole-genome sequencing: *Elizabethkingia endophytica* Kämpfer et al. 2015 is a later subjective synonym of *Elizabethkingia anophelis* Kämpfer et al. 2011. Int. J. Syst. Evol. Microbiol..

[29] Lin J.-N., Lai C.-H., Yang C.-H., Huang Y.-H., Lin H.-F., Lin H.-H. (2017). Comparison of four automated microbiology systems with 16S rRNA gene sequencing for identification of *Chryseobacterium* and *Elizabethkingia* species. Sci. Rep..

[30] Han M.-S., Kim H., Lee Y., Kim M., Ku N.S., Choi J.Y., Yong D., Jeong S.H., Lee K., Chong Y. (2017). Relative prevalence and antimicrobial susceptibility of clinical isolates of *Elizabethkingia* species based on 16S rRNA gene sequencing. J. Clin. Microbiol..

[31] Cheng Y.-H., Perng C.-L., Jian M.-J., Cheng Y.-H., Lee S.-Y., Sun J.-R., Shang H.-S. (2019). Multicentre study evaluating matrix-assisted laser desorption ionization-time of flight mass spectrometry for identification of clinically isolated *Elizabethkingia* species and analysis of antimicrobial susceptibility. Clin. Microbiol. Infect..

[32] Janda J.M., Abbott S.L. (2007). 16S rRNA gene sequencing for bacterial identification in the diagnostic laboratory: Pluses, perils, and pitfalls. J. Clin. Microbiol..

[33] Adékambi T., Drancourt M., Raoult D. (2009). The *rpoB* gene as a tool for clinical microbiologists. Trends Microbiol..

[34] Holmes B., Steigerwalt A.G., Nicholson A.C. (2013). DNA-DNA hybridization study of strains of *Chryseobacterium*, *Elizabethkingia* and *Empedobacter* and of other usually indole-producing non-fermenters of CDC groups IIc, IIe, IIh and IIi, mostly from human clinical sources, and proposals of *Chryseobacterium bernardetii* sp. nov., *Chryseobacterium carnis* sp. nov., *Chryseobacterium lactis* sp. nov., *Chryseobacterium nakagawai* sp. nov. and *Chryseobacterium taklimakanense* comb. nov. Int. J. Syst. Evol. Microbiol..

[35] Pei A.Y., Oberdorf W.E., Nossa C.W., Agarwal A., Chokshi P., Gerz E.A., Jin Z., Lee P., Yang L., Poles M. (2010). Diversity of 16S rRNA genes within individual prokaryotic genomes. Appl. Environ. Microbiol..

[36] Walsh D.A., Bapteste E., Kamekura M., Doolittle W.F. (2004). Evolution of the RNA polymerase B’ subunit gene (*rpoB’*) in *Halobacteriales*: A complementary molecular marker to the SSU rRNA gene. Mol. Biol. Evol..

[37] Rowland G.C., Aboshkiwa M., Coleman G. (1993). Comparative sequence analysis and predicted phylogeny of the DNA-dependent RNA polymerase beta subunits of *Staphylococcus aureus* and other eubacteria. Biochem. Soc. Trans..

[38] Chew K.L., Cheng B., Lin R.T.P., Teo J.W.P. (2018). *Elizabethkingia anophelis* is the dominant *Elizabethkingia* species found in blood cultures in Singapore. J. Clin. Microbiol..

[39] Kelly A.J., Karpathy S.E., Gulvik C.A., Ivey M.L., Whitney A.M., Bell M.E., Nicholson A.C., Humrighouse B.H., McQuiston J.R. (2019). A real-time multiplex PCR assay for detection of *Elizabethkingia* species, and differentiating between *E. anophelis* and *E. meningoseptica*. J. Clin. Microbiol..

[40] Goris J., Konstantinidis K.T., Klappenbach J.A., Coenye T., Vandamme P., Tiedje J.M. (2007). DNA-DNA hybridization values and their relationship to whole-genome sequence similarities. Int. J. Syst. Evol. Microbiol..

[41] Richter M., Rosselló-Móra R. (2009). Shifting the genomic gold standard for the prokaryotic species definition. Proc. Natl. Acad. Sci. USA.

[42] Konstantinidis K.T., Tiedje J.M. (2005). Genomic insights that advance the species definition for prokaryotes. Proc. Natl. Acad. Sci. USA.

[43] Lin J.-N., Lai C.-H., Yang C.-H., Huang Y.-H., Lin H.-H. (2019). Genomic features, comparative genomics, and antimicrobial susceptibility patterns of *Elizabethkingia bruuniana*. Sci. Rep..

[44] Lin J.-N., Lai C.-H., Yang C.-H., Huang Y.-H., Lin H.-H. (2017). Genomic features, phylogenetic relationships, and comparative genomics of *Elizabethkingia anophelis* strain EM361-97 isolated in Taiwan. Sci. Rep..

[45] Eriksen H.B., Gumpert H., Faurholt C.H., Westh H. (2017). Determination of *Elizabethkingia* diversity by MALDI-TOF mass spectrometry and whole-genome sequencing. Emerg. Infect. Dis..

[46] Lin J.-N., Lai C.-H., Yang C.-H., Huang Y.-H., Lin H.-H. (2018). Complete genome sequence of *Elizabethkingia miricola* strain EM798-26 isolated from the blood of a cancer patient. Genome Announc..

[47] Breurec S., Criscuolo A., Diancourt L., Rendueles O., Vandenbogaert M., Passet V., Caro V., Rocha E.P.C., Touchon M., Brisse S. (2016). Genomic epidemiology and global diversity of the emerging bacterial pathogen *Elizabethkingia anophelis*. Sci. Rep..

[48] Da Silva P.S.L., Pereira G.H. (2013). *Elizabethkingia meningoseptica*: Emergent bacteria causing pneumonia in a critically ill child. Pediatr. Int..

[49] Lin P.-Y., Chu C., Su L.-H., Huang C.-T., Chang W.-Y., Chiu C.-H. (2004). Clinical and microbiological analysis of bloodstream infections caused by *Chryseobacterium meningosepticum* in nonneonatal patients. J. Clin. Microbiol..

[50] Lin Y.-T., Chiu C.-H., Chan Y.-J., Lin M.-L., Yu K.-W., Wang F.-D., Liu C.-Y. (2009). Clinical and microbiological analysis of *Elizabethkingia meningoseptica* bacteremia in adult patients in Taiwan. Scand. J. Infect. Dis..

[51] Hung P.-P., Lin Y.-H., Lin C.-F., Liu M.-F., Shi Z.-Y. (2008). *Chryseobacterium meningosepticum* infection: Antibiotic susceptibility and risk factors for mortality. J. Microbiol. Immunol. Infect..

[52] Hsu M.-S., Liao C.-H., Huang Y.-T., Liu C.-Y., Yang C.-J., Kao K.-L., Hsueh P.-R. (2011). Clinical features, antimicrobial susceptibilities, and outcomes of *Elizabethkingia meningoseptica* (*Chryseobacterium meningosepticum*) bacteremia at a medical center in Taiwan, 1999–2006. Eur. J. Clin. Microbiol. Infect. Dis..

[53] Huang Y.-C., Huang Y.-W., Lin Y.-T., Wang F.-D., Chan Y.-J., Yang T.-C. (2017). Risk factors and outcome of levofloxacin-resistant *Elizabethkingia meningoseptica* bacteraemia in adult patients in Taiwan. Eur. J. Clin. Microbiol. Infect. Dis..

[54] Huang Y.-C., Lin Y.-T., Wang F.-D. (2018). Comparison of the therapeutic efficacy of fluoroquinolone and non-fluoroquinolone treatment in patients with *Elizabethkingia meningoseptica* bacteraemia. Int. J. Antimicrob. Agents.

[55] Chen W.-C., Chen Y.-W., Ko H.-K., Yu W.-K., Yang K.-Y. (2018). Comparisons of clinical features and outcomes between *Elizabethkingia meningoseptica* and other glucose non-fermenting Gram-negative bacilli bacteremia in adult ICU patients. J. Microbiol. Immunol. Infect..

[56] Huang Y.-C., Wu P.-F., Lin Y.-T., Wang F.-D. (2019). Comparison of clinical characteristics of bacteremia from *Elizabethkingia meningoseptica* and other carbapenem-resistant, non-fermenting Gram-negative bacilli at a tertiary medical center. J. Microbiol. Immunol. Infect..

[57] Rastogi N., Mathur P., Bindra A., Goyal K., Sokhal N., Kumar S., Sagar S., Aggarwal R., Soni K.D., Tandon V. (2016). Infections due to *Elizabethkingia meningoseptica* in critically injured trauma patients: A seven-year study. J. Hosp. Infect..

[58] Lin P.-Y., Chen H.-L., Huang C.-T., Su L.-H., Chiu C.-H. (2010). Biofilm production, use of intravascular indwelling catheters and inappropriate antimicrobial therapy as predictors of fatality in *Chryseobacterium meningosepticum* bacteraemia. Int. J. Antimicrob. Agents.

[59] Bloch K.C., Nadarajah R., Jacobs R. (1997). *Chryseobacterium meningosepticum*: An emerging pathogen among immunocompromised adults. Report of 6 cases and literature review. Medicine (Baltimore).

[60] Green O., Murray P., Gea-Banacloche J.C. (2008). Sepsis caused by *Elizabethkingia miricola* successfully treated with tigecycline and levofloxacin. Diagn. Microbiol. Infect. Dis..

[61] Colapietro M., Endimiani A., Sabatini A., Marcoccia F., Celenza G., Segatore B., Amicosante G., Perilli M. (2016). BlaB-15, a new BlaB metallo-β-lactamase variant found in an *Elizabethkingia miricola* clinical isolate. Diagn. Microbiol. Infect. Dis..

[62] Opota O., Diene S.M., Bertelli C., Prod’hom G., Eckert P., Greub G. (2017). Genome of the carbapenemase-producing clinical isolate *Elizabethkingia miricola* EM_CHUV and comparative genomics with *Elizabethkingia meningoseptica* and *Elizabethkingia anophelis*: Evidence for intrinsic multidrug resistance trait of emerging pathogens. Int. J. Antimicrob. Agents.

[63] Zdziarski P., Paściak M., Rogala K., Korzeniowska-Kowal A., Gamian A. (2017). *Elizabethkingia miricola* as an opportunistic oral pathogen associated with superinfectious complications in humoral immunodeficiency: A case report. BMC Infect. Dis..

[64] Kenna D.T.D., Fuller A., Martin K., Perry C., Pike R., Burns P.J., Narayan O., Wilkinson S., Hill R., Woodford N. (2018). *rpoB* gene sequencing highlights the prevalence of an *E. miricola* cluster over other *Elizabethkingia* species among UK cystic fibrosis patients. Diagn. Microbiol. Infect. Dis..

[65] Lau S.K.P., Wu A.K.L., Teng J.L.L., Tse H., Curreem S.O.T., Tsui S.K.W., Huang Y., Chen J.H.K., Lee R.A., Yuen K.-Y. (2015). Evidence for *Elizabethkingia anophelis* transmission from mother to infant, Hong Kong. Emerg. Infect. Dis..

[66] Fraser S.L., Jorgensen J.H. (1997). Reappraisal of the antimicrobial susceptibilities of *Chryseobacterium* and *Flavobacterium* species and methods for reliable susceptibility testing. Antimicrob. Agents Chemother..

[67] Lin J.-N., Lai C.-H., Yang C.-H., Huang Y.-H. (2019). *Elizabethkingia bruuniana* infections in humans, Taiwan, 2005–2017. Emerg Infect Dis..

[68] Tai I.-C., Liu T.-P., Chen Y.-J., Lien R.-I., Lee C.-Y., Huang Y.-C. (2017). Outbreak of *Elizabethkingia meningoseptica* sepsis with meningitis in a well-baby nursery. J. Hosp. Infect..

[69] Chen S., Soehnlen M., Walker E.D. (2016). Genome sequence of *Elizabethkingia meningoseptica* EM1, isolated from a patient with a bloodstream infection. Genome Announc..

[70] Jian M.-J., Cheng Y.-H., Perng C.-L., Shang H.-S. (2018). Molecular typing and profiling of topoisomerase mutations causing resistance to ciprofloxacin and levofloxacin in *Elizabethkingia* species. Peer J..

[71] Li Y., Liu Y., Chew S.C., Tay M., Salido M.M.S., Teo J., Lauro F.M., Givskov M., Yang L. (2015). Complete genome sequence and transcriptomic analysis of the novel pathogen *Elizabethkingia anophelis* in response to oxidative stress. Genome Biol. Evol..

[72] Jian M.-J., Cheng Y.-H., Chung H.-Y., Cheng Y.-H., Yang H.-Y., Hsu C.-S., Perng C.-L., Shang H.-S. (2019). Fluoroquinolone resistance in carbapenem-resistant *Elizabethkingia anophelis*: Phenotypic and genotypic characteristics of clinical isolates with topoisomerase mutations and comparative genomic analysis. J. Antimicrob. Chemother..

